# Effects of occipital-atlas stabilization on the upper cervical spine rotation combinations: an in vitro study

**DOI:** 10.1038/s41598-023-30512-3

**Published:** 2023-03-02

**Authors:** César Hidalgo-García, Ana I. Lorente, Carlos López-de-Celis, María Orosia Lucha-López, Jacobo Rodríguez-Sanz, Mario Maza-Frechín, José Miguel Tricás-Moreno, John Krauss, Albert Pérez-Bellmunt

**Affiliations:** 1 Unidad de Investigación en Fisioterapia, Facultad de Ciencias de la Salud de la Universidad de Zaragoza, C/ Domingo Miral S/N, 50009 Zaragoza, Spain; 2 Impact Laboratory, Aragon Institute of Engineering Research, Universidad de Zaragoza, Alcañiz, Spain; 3 ACTIUM Anatomy Group, Faculty of Medicine and Health Sciences, Universitat Internacional de Catalunya, Sant Cugat del Vallès, Spain; 4 Fundació Institut Universitari per a la recerca a l’Atenció Primaria de Salut Jordi Gol i, Barcelona, Spain; 5 School of Health Sciences, Oakland University, Rochester, MI USA

**Keywords:** Anatomy, Health care

## Abstract

The purpose of this study is to compare axial rotation range of motion for the upper cervical spine during three movements: axial rotation, rotation + flexion + ipsilateral lateral bending and rotation + extension + contralateral lateral bending before and after occiput-atlas (C0–C1) stabilization. Ten cryopreserved C0–C2 specimens (mean age 74 years, range 63–85 years) were manually mobilized in 1. axial rotation, 2. rotation + flexion + ipsilateral lateral bending and 3. rotation + extension + contralateral lateral bending without and with a screw stabilization of C0–C1. Upper cervical range of motion and the force used to generate the motion were measured using an optical motion system and a load cell respectively. The range of motion (ROM) without C0–C1 stabilization was 9.8° ± 3.9° in right rotation + flexion + ipsilateral lateral bending and 15.5° ± 5.9° in left rotation + flexion + ipsilateral lateral bending. With stabilization, the ROM was 6.7° ± 4.3° and 13.6° ± 5.3°, respectively. The ROM without C0–C1 stabilization was 35.1° ± 6.0° in right rotation + extension + contralateral lateral bending and 29.0° ± 6.5° in left rotation + extension + contralateral lateral bending. With stabilization, the ROM was 25.7° ± 6.4° (*p* = 0.007) and 25.3° ± 7.1°, respectively. Neither rotation + flexion + ipsilateral lateral bending (left or right) or left rotation + extension + contralateral lateral bending reached statistical significance. ROM without C0–C1 stabilization was 33.9° ± 6.7° in right rotation and 28.0° ± 6.9° in left rotation. With stabilization, the ROM was 28.5° ± 7.0° (*p* = 0.005) and 23.7° ± 8.5° (*p* = 0.013) respectively. The stabilization of C0–C1 reduced the upper cervical axial rotation in right rotation + extension + contralateral lateral bending and right and left axial rotations; however, this reduction was not present in left rotation + extension + contralateral lateral bending or both combinations of rotation + flexion + ipsilateral lateral bending.

## Introduction

The specialized anatomy of the upper cervical spine (UCS), comprised of the occipital-atlas (C0–C1) and atlas-axis (C1–C2) segments, produces complex three-dimensional movements and subsequently is the most mobile region of the spine^1^. These segments produce approximately 60% of the cervical axial rotation^1,2^, and the greatest axial rotation of any segments in the spine^3^. UCS axial rotation is mainly restrained by the alar ligament system, connecting the occipital bone to the odontoid process of C2 bilaterally^4^.

The generally accepted range of movement during unilateral UCS axial rotation is 40–45°^5^. C1–C2 produces the largest magnitude of axial rotation^6^ with C0–C1 often disregarded due to its minimal contribution to rotation^2,6–9^.

Using a mathematical model, Boszczyk et al. (2012) could not account for the 40–45° of UCS axial rotation range of motion (ROM) with the alar ligaments intact using the arthrokinematics of C1–C2 alone^8^. They emphasized the role of coupled motions in the frontal and sagittal planes associated with UCS axial rotation as a possible explanation for the amount of C1–C2 axial rotation ROM^8^. Their rationale was that the tightening of alar ligaments would impact the available ROM in the different coupled movements associated with UCS axial rotation. The combination of axial rotation and contralateral lateral bending is considered the coupled movement for the UCS rotation^2,5,6,9–12^. Coupled rotation + extension + contralateral lateral bending, and axial rotation alone, showed larger UCS rotation ROM than simultaneous flexion, axial rotation and ipsilateral lateral bending) in an in vitro study^13^. Rotation in flexion is associated to maximal tightening of the alar ligaments^14^.

However, the model of Boszczyk et al. (2012), considered the base of the skull and the atlas a functional unit in respect to axial rotation, disregarding any motion in C0–C1^8^. However, Hidalgo et al.^15^ demonstrated using an in vitro design that UCS rotation was reduced by 15% and resistance to mobilization was increased when C0–C1 was stabilized compared to when C0–C1 was allowed to rotate freely^16^. They proposed that C0–C1 kinematics could be related to the tightening of the alar ligaments and therefore to UCS axial rotation^16^. The relationship of C0–C1 and the tightening of the alar ligaments is supported by the insertion of the alar ligaments medially and close to the atlanto-occipital joint capsules^4^. The effect of C0–C1 kinematics on the ROM of different combined movements of UCS axial rotation is unknown.

The purpose of this in vitro study is to compare the ROM of the upper cervical spine in axial rotation, rotation + flexion + ipsilateral lateral bending combination, and rotation + extension + contralateral lateral bending combination before and after C0–C1 stabilization. We hypothesized that C0–C1 stabilization: (a) will reduce UCS axial rotation in all UCS rotation combinations, and (b) the ROM reduction will be larger in the rotation + extension + contralateral lateral bending combination.

## Methods

### Sample

Ten cervical spines and heads from cryopreserved cadavers (9 males, 1 female, mean age: 74 years, range: 63–85 years) were examined. All specimens were visually checked for any anatomical anomaly that would influence ROM. All specimens were donated to Universitat Internacional de Catalunya (UIC) and specimens were not procured from prisoners. Informed consent was obtained from the organ donor for donating the cadaver body for researching purposes. The study was approved by a Research Ethics Committee from UIC-Barcelona (Ref. CBAS-2017-03) and all methods were carried out in accordance with relevant guidelines and regulations.

### Anatomical and biomechanical procedure

This study is a secondary analysis of Hidalgo-García et al.^16^, which measured upper cervical mobility in the cardinal planes before and after the stabilization of C0–C1, and Lorente et al.^13^, which measured the combined upper cervical rotation movement with and without unilateral transection of the alar ligament. All specimens were stored at − 14 °C and thawed to room temperature 24 h before testing. The preparation of the C0–C2 specimens started with the disarticulation of C2–C3 and the removal of all myofascial tissues without disrupting the ligamentous tissues. In order to expose the alar ligaments, the cranial posterior third of the skull and the brain, brainstem, spinal cord, dura and part of the tectorial membrane were removed. Finally, the mandible and the upper maxilla were removed in order to attach the motion measurement sensors. After the anatomical dissection, a metallic handlebar was attached to the skull by three points: one in each auditory canal and one at the top of the head.

**Figure 1:**
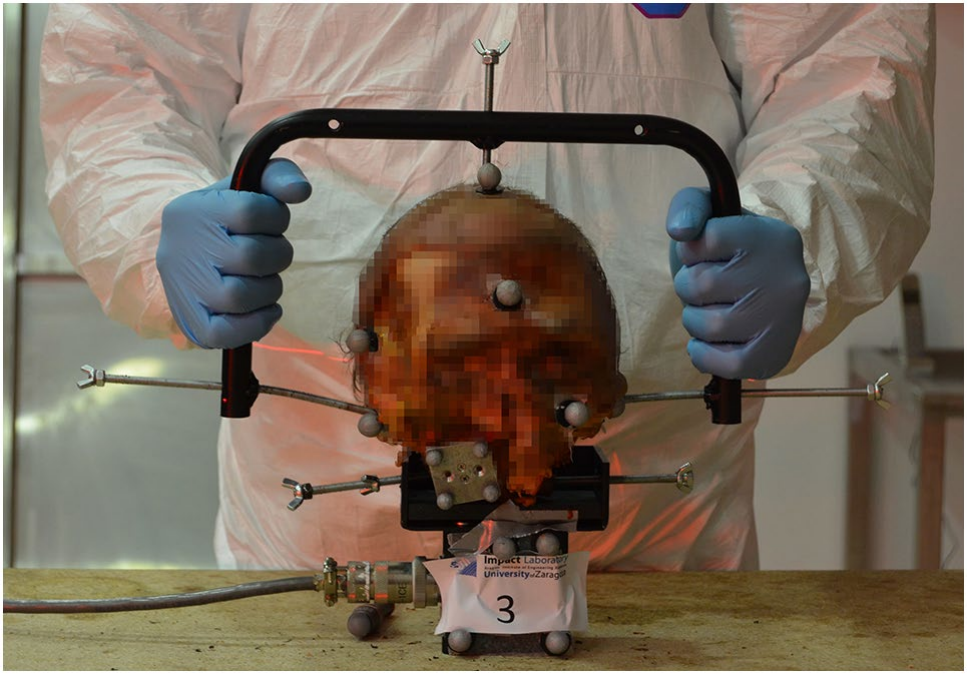
C0–C2 specimen: starting set up of the test.

The C2 vertebra was screwed to a metallic support that was fixed to the load cell (MC3-6-100 Force and Torque Sensor, Advanced Mechanical Technology Inc., Watertown, USA), which measured the torque required to generate the movement in the transverse plane. The specimen was kept in an upright position and the three anatomical planes and the three axes of the load cell were aligned (Fig. 1). The tester moved the skull through the handlebar from the posterior part of the skull. The handlebar avoided touching the motion measurement sensors and obscuring them from the cameras.

The force applied by the tester when moving the specimens was converted to Newtons from the torque measured by the load cell. The reason for providing the values in Newtons is to facilitate the understanding from a clinical point of view. The distance between the handlebar and the estimated axis of rotation was used for this calculation: 150 mm for the rotation in the transverse plane (the half of the metallic handlebar width). This measurement was approximated to 150 mm due to the small variations shown by the instantaneous axis of rotation within the individual segments^17,18^. The force values reported in Newtons represent the total load from both hands of the tester in axial rotation.

The head was aligned in the neutral position before each test. Two lines were marked on the head: the Frankfurt horizontal plane was laterally marked (through the infraorbital foraminae and the external auditory meati), and a vertical line on the center of the face (from the center of the chin to the center of the forehead). Two red light lasers calibrated horizontally and vertically were aligned with these two horizontal and vertical lines.

The motion of the head and the two upper cervical vertebrae (C1 and C2) was tracked by an optical motion capture system (TS Series, Vicon, Oxford, UK) with four cameras. Retroreflective spherical markers were directly placed on the head (Fig. 1). Four markers were attached on a metallic plate, which was screwed to C1. The C1 motion was tracked to assess the C0–C1 motion pre- and post-screw fixation, and its metallic plate did not influence the intersegmental motion. Finally, the markers for C2 were fixed on the load cell, where C2 was attached.

**Figure 2:**
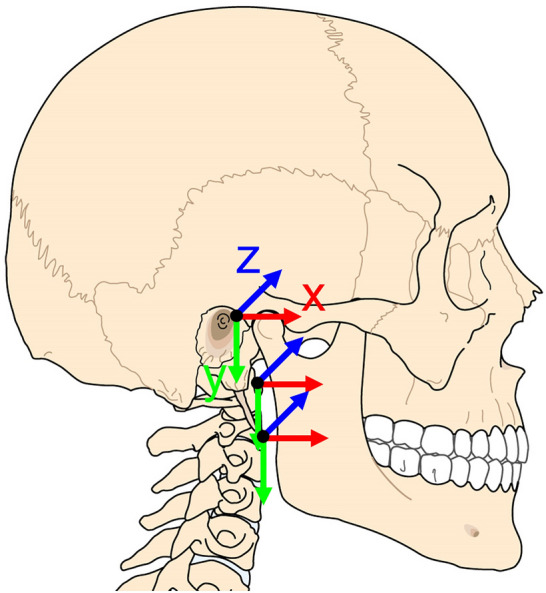
Local coordinate systems of the head, C1 and C2.

A 3D measuring device (FaroArm, FARO Technologies, Lake Mary, FL, USA) was used to calculate the local coordinate systems of the head, C1 and C2. The FaroArm measured the anatomical landmarks on the (1) skull: right and left auditory meati and right infraorbital foraminae, (2) C1: symmetrical right and left landmarks on the transverse processes, and anterior and posterior tubercles, and (3) C2: symmetrical right and left landmarks on the transverse processes, lowest anterior central point on the body, and lowest central point on the spinous process. With this FaroArm measurements, the coordinate systems had the X-axis pointing forward, the Y-axis pointing from left to right, and completing a right-hand-oriented coordinate system, the Z-axis pointed downwards (Fig. 2). To convert this axes orientation to the ISB recommendation: the + X orientation does not differ, while + Y would be our − Z and + Z would be our + Y^19^. This only differ in our results by switching the sign between right and left axial rotation. The coordinate system (orientation and origin) of the skull was defined following Slykhouse et al.^20^. The coordinate systems for C1 and C2 have the orientation shown in Slykhouse et al.^20^ and they were defined with the landmarks mentioned above: the points on the right and left side of the vertebrae and the anterior and posterior references. The origin in C1 and C2 was located at the anterior point landmark. Apart from the anatomical landmarks, FaroArm also digitized the Vicon markers. By doing this, the Vicon and FaroArm measurements allowed to measure the motion of each segment. The transformation from the Vicon data to the local coordinate systems was calculated following Shaw et al.^21^.

The installation of a manual trigger made it possible the synchronization of the data collection from both the load cell and the motion capture system. Both records started simultaneously and ended after a pre-defined time of 15 s (rotation + flexion + ipsilateral lateral bending) or 20 s (axial rotation and rotation + extension + contralateral lateral bending). The pre-defined time was always long enough to reach the full-ROM. To analyze the motion among all the specimens, motion data was extracted at three levels of the load curve: 1N, 2N and maximum load.

Each specimen was moved in right and left rotation with the same procedure as in Lorente et al.^13^:

1. Axial rotation,
2. Rotation with extension and contralateral lateral bending (motion in the three anatomical planes at the same time),
3. Rotation with flexion and ipsilateral lateral bending (motion in the three anatomical planes at the same time).

The same order (1–2–3) was followed for all the specimens. Before measuring the (1–2–3) mobilization sequence, the head was mobilized three times (full-ROM) in flexion–extension and lateral bending, and two times in axial rotation. A third mobilization in axial rotation was the one analyzed. After performing these measurements with normal configuration (without fixation), an occipital-atlas screw stabilization was performed. The occipital entry point of the screw was 5 mm lateral to the foramen magnum, penetrating into the lateral mass of atlas. The stabilization procedure is described in detail in Hidalgo-García et al.^16^. Next, the same measurements were performed in the same sequence (1–2–3). The axial rotation of the skull with respect to the C2 local coordinate system was obtained following the equations described in Paul^22^.

A researcher with more than 15 years of clinical experience treating patients with upper cervical impairments induced all movements manually until a marked resistance. To prevent dehydration and ensure physiological properties, the dissection room was maintained with a temperature between 17.0 and 17.8 °C, and a humidity between 47 and 52%. Temperature and humidity play an important role when testing spine specimens: temperature should be controlled and protection against drying out should be provided to the specimens^23^.

SPSS 23.0 (IBM, Armonk, New York) was used to conduct the statistical analysis. The mean and standard deviation were calculated for each variable. A Wilcoxon Signed Rank Test was performed to analyze intergroup differences, with a significance level set at *p* < 0.05.

### Ethical approval

Research Ethics Committee from UIC-Barcelona. Ref. CBAS-2017-03.

## Results

Table 1 contains the ROM of axial rotation, rotation + flexion + ipsilateral lateral bending, and rotation + extension + contralateral lateral bending recorded for each specimen when the applied forces were 1N, and 2N during the motion and the maximum force (F. Max) with its range of motion (ROM Max). The table shows the values for all the specimens before and after the stabilization of C0–C1.

**Table 1:** ROM of axial rotation, rotation + flexion + ipsilateral lateral bending (Rot flex) and rotation + extension + contralateral lateral bending (Rot ext) recorded for each specimen when the applied forces were 1N, 2N during the motion and the maximum force (F. Max) with its range of motion (ROM Max). The table shows the values for all the specimens before (normal) and after the stabilization of C0–C1 (C0C1 stab).

Test		Test type								
			Right				Left			
			1 N	2 N	F. Max (N)	ROM Max (°)	1 N	2 N	F. Max (N)	ROM Max (°)
1	**Normal**	**Rot flex**			0.2	3.6	15.9°		1.9	20.9
		**Rotation**	26.1°	30.0°	2.0	30.8	33.6°	36.0°	2.9	38.0
		**Rot ext**	23.8°	28.5°	5.7	34.1	32.0°	34.9°	3.1	37.7
	**C0C1 stab**	**Rot flex**			0.0	4.3			0.0	13.1
		**Rotation**	16.6°	23.5°	3.5	27.3	26.1°	34.9°	2.1	41.9
		**Rot ext**	15.0°	21.1°	4.5	28.2	5.3°	31.6°	4.8	39.3
	***Difference***	**Rot flex**			− 0.2	0.7			− 1.9	− 7.7
		**Rotation**	− 9.6°	− 6.5°	1.4	− 3.5	− 7.5°	− 1.1°	− 0.8	3.9
		**Rot ext**	− 8.8°	− 7.5°	− 1.3	− 5.9	− 26.7°	− 3.4°	1.6	1.7
2	**Normal**	**Rot flex**	3.9°		1.3	12			0.2	23.7
		**Rotation**	5.7°	32.6°	2.5	33.5	38.5°		1.3	39.0
		**Rot ext**	0.3°	13.3°	5.5	35.1	32.3°	36.7°	3.5	41.2
	**C0C1 stab**	**Rot flex**			0.9	15.9			0.4	18.2
		**Rotation**	0.4°	30.0°	2.7	31.9			11.0	31.9
		**Rot ext**	1.8°	19.8°	2.6	27.0	27.4°		1.8	30.0
	***Difference***	**Rot flex**			− 0.4	3.9			0.2	− 5.5
		**Rotation**	− 5.2°	− 2.6°	0.2	− 1.6			− 0.3	− 7.1
		**Rot ext**	1.5°	6.5°	− 2.9	− 8.9	− 4.9°		− 1.6	− 11.2
3	**Normal**	**Rot flex**	0.6°	2.2°	3.3	4.6	4.8°		1.8	5.4
		**Rotation**	0.0°	7.5°	3.4	24.8	13.6°	16.4°	2.5	17.6
		**Rot ext**	19.9°	24.6°	2.4	25.8	21.3°		1.6	22.0
	**C0C1 stab**	**Rot flex**	− 0.1°	0.8°	3.3	1.3	2.2°	4.5°	2.2	5.1
		**Rotation**		1.0°	3.9	19.8			0.7	17.3
		**Rot ext**		16.4°	3.3	21.8	23.1°		1.9	24.7
	***Difference***	**Rot flex**	− 0.7°	− 1.4°	0.0	− 3.3	− 2.6°		0.4	− 0.3
		**Rotation**		− 6.5°	0.5	− 5.0			− 1.8	− 0.3
		**Rot ext**		− 8.2°	0.9	− 4.0	1.8°		0.3	2.7
4	**Normal**	**Rot flex**			0.8	9.1	9.6°		1.4	11.3
		**Rotation**	38.0°	42.7°	3.8	43.8	15.6°	20.7°	4.3	25.4
		**Rot ext**	33.6°	40.4°	2.6	42.4	13.1°	19.9°	6.6	29.0
	**C0C1 stab**	**Rot flex**			0.4	2.9	2.3°		1.8	4.7
		**Rotation**	0.4°	34.9°	4.2	40.9	7.1°	11.4°	3.1	15.6
		**Rot ext**	0.3°	3.2°	4.3	37.0	0.8°	11.5°	4.5	21.1
	***Difference***	**Rot flex**			− 0.4	− 6.2	− 7.3°		0.4	− 6.6
		**Rotation**	− 37.6°	− 7.8°	0.4	− 2.9	− 8.6°	− 9.3°	− 1.3	− 9.7
		**Rot ext**	− 33.3°	− 37.2°	1.7	− 5.4	− 12.4°	− 8.4°	− 2.1	− 7.9
5	**Normal**	**Rot flex**	10.1°		1.2	11.5	9.6°		2.0	12.1
		**Rotation**	21.3°	22.1°	3.1	24.6	12.2°	14.5°	5.3	21.1
		**Rot ext**	11.6°	19.5°	5.0	26.6	4.8°	11.2°	6.6	21.4
	**C0C1 stab**	**Rot flex**			0.3	6.8	6.6°	9.2°	3.9	11.9
		**Rotation**	15.0°	21.3°	3.7	24.5	7.9°	11.0°	5.0	15.2
		**Rot ext**	5.2°	13.2°	2.4	15.3	0.4°	9.1°	4.8	17.4
	***Difference***	**Rot flex**			− 0.9	− 4.7	− 3.0°		1.9	− 0.3
		**Rotation**	− 6.3°	− 0.8°	0.6	− 0.1	− 4.3°	− 3.6°	− 0.3	− 6.0
		**Rot ext**	− 6.4°	− 6.3°	− 2.7	− 11.3	− 4.4°	− 2.1°	− 1.8	− 44
**6**	**Normal**	**Rot flex**			0.4	7.1	9.6°		1.1	9.8
		**Rotation**	26.7°	29.2°	2.8	30.5	15.1°	18.8°	4.6	22.1
		**Rot ext**	24.9°	28.9°	3.7	32.0	15.7°	19.0°	4.6	23.0
	**C0C1 stab**	**Rot flex**			0.8	5.6	2.4°	7.4°	3.6	12.5
		**Rotation**	1.3°	8.9°	5.7	23.6	3.5°	7.5°	6.4	16.9
		**Rot ext**	1.7°	5.5°	5	25.4	0.4°	3.6°	5.8	14.2
	***Difference***	**Rot flex**			0.4	− 1.5	− 7.2°		2.6	2.7
		**Rotation**	− 25.4°	− 20.3°	2.8	− 6.8	− 11.6°	− 11.3°	1.8	− 5.2
		**Rot ext**	− 23.2°	− 23.5°	1.3	− 6.6	− 15.2°	− 15.4°	1.2	− 8.8
7	**Normal**	**Rot flex**	10.6°		1.4	12.6	14.4°		1.2	15.9
		**Rotation**	22.7°	31.0°	4.0	36.9	21.5°	26.3°	2.9	28.4
		**Rot ext**	32.9°	36.3°	2.9	38.8	17.8°	25.0°	3.1	28.7
	**C0C1 stab**	**Rot flex**			0.8	9.3			0.6	16.3
		**Rotation**	19.6°	24.9°	3.8	32.7	18.0°		2.0	21.1
		**Rot ext**	19.6°	25.5°	3.1	30.7	11.8°	18.5°	3.5	23.1
	***Difference***	**Rot flex**			− 0.6	− 3.3			− 0.6	0.4
		**Rotation**	− 3.1°	− 6.0°	− 0.2	− 4.1	− 3.6°		− 0.9	− 7.3
		**Rot ext**	− 13.3°	− 10.8°	0.2	− 8.1	− 6.0°	− 6.6°	0.3	− 5.5
8	**Normal**	**Rot flex**	7.0°		1.7	14.7	7.7°	13.0°	2.5	15.3
		**Rotation**	30.0°	34.3°	2.8	36.5	23.2°	26.5°	4.8	30.8
		**Rot ext**	34.9°	37.4°	3.0	41.0	25.9°	27.9°	4.6	31.5
	**C0C1 stab**	**Rot flex**	2.5°		1.9	6.0	11.6°		1.5	16.5
		**Rotation**	11.4°	19.2°	3.9	26.5	19.8°	24.3°	3.1	26.3
		**Rot ext**	20.1°	24.4°	4.6	30.3	24.8°	26.3°	3.2	28.0
	***Difference***	**Rot flex**	− 4.5°		0.2	− 8.7	4.0°		− 11.0	1.2
		**Rotation**	− 18.6°	− 15.2°	1.0	− 10.0	− 3.4°	− 2.3°	− 1.7	− 4.5
		**Rot ext**	− 14.9°	− 13.0°	1.6	− 10.7	− 1.7°	− 1.6°	− 1.4	− 3.5
9	**Normal**	**Rot flex**	5.9°		1.7	8.4	5.3°	12.5°	3.7	19.4
		**Rotation**	16.1°	31.1°	5.5	43.5	18.9°	23.7°	4.2	28.0
		**Rot ext**	34.7°	39.4°	3.0	41.9	16.8°	20.9°	4.9	27.5
	**C0C1 stab**	**Rot flex**	5.0°	10.7°	2.1	11.0	5.0°	15.1°	2.7	17.5
		**Rotation**	18.7°	28.9°	3.8	36.8	18.3°	22.0°	2.2	22.5
		**Rot ext**			0.7	21.9	21.9°		1.8	24.7
	***Difference***	**Rot flex**	− 0.9°		0.4	2.6	− 0.3°	2.6°	− 1.0	− 1.8
		**Rotation**	2.6°	− 2.2°	− 1.6	− 6.7	− 0.5°	− 1.7°	− 2.0	− 5.5
		**Rot ext**			− 2.3	− 20.1	5.1°		− 3.1	− 2.8
10	**Normal**	**Rot flex**	12.0°		1.3	14.4	16.7°	20.3°	2.5	21.6
		**Rotation**	24.3°	30.1°	3.5	33.7	23.4°	26.0°	5.5	29.5
		**Rot ext**	28.8°	32.7°	2.3	33.5	24.9°	26.7°	2.7	27.6
	**C0C1 stab**	**Rot flex**	2.4°		1.5	4.0	0.8°	17.3°	2.7	20.1
		**Rotation**	0.8°	13.6°	3.8	20.5	16.9°	22.5°	4.5	27.6
		**Rot ext**	2.5°	13.9°	3.3	19.1	9.7°	23.3°	6.2	30.1
	***Difference***	**Rot flex**	− 9.6°		0.2	− 10.4	− 15.8°	− 3.0°	0.2	− 1.5
		**Rotation**	− 23.4°	− 16.5°	0.3	− 13.2	− 6.6°	− 3.5°	− 0.9	− 1.9
		**Rot ext**	− 26.3°	− 18.8°	0.9	− 14.4	− 15.2°	− 3.4°	3.5	2.6

### Axial rotation mobility

During right axial rotation, the end ROM without C0–C1 stabilization was 33.9° ± 6.7°, with an average maximum force of 3.4N ± 0.9N. Following C0–C1 stabilization, the specimens demonstrated a statistically significant average reduction of 5.4° ± 3.9° (*p* = 0.005) at end range with an average maximum force of 3.9N ± 0.7N. This reduction of right axial rotation was also seen with standardized forces of 1N and 2N. During left axial rotation, the average end ROM without C0–C1 stabilization was 28.0° ± 6.9°, with an average maximum force of 3.8N ± 1.4N. All specimens demonstrated a statistically significant reduction in left rotation ROM following the stabilization of C0–C1 of 4.3° ± 3.9° (*p* = 0.013) with an average maximum force of 3.0N ± 1.8N at end-range. This reduction was also statistically significant during all standardized forces. However, not all specimens showed a reduction and specimen #1 showed an increase of left axial rotation after C0–C1 stabilization.

### Rotation + extension + contralateral lateral bending mobility

**Figure 3:**
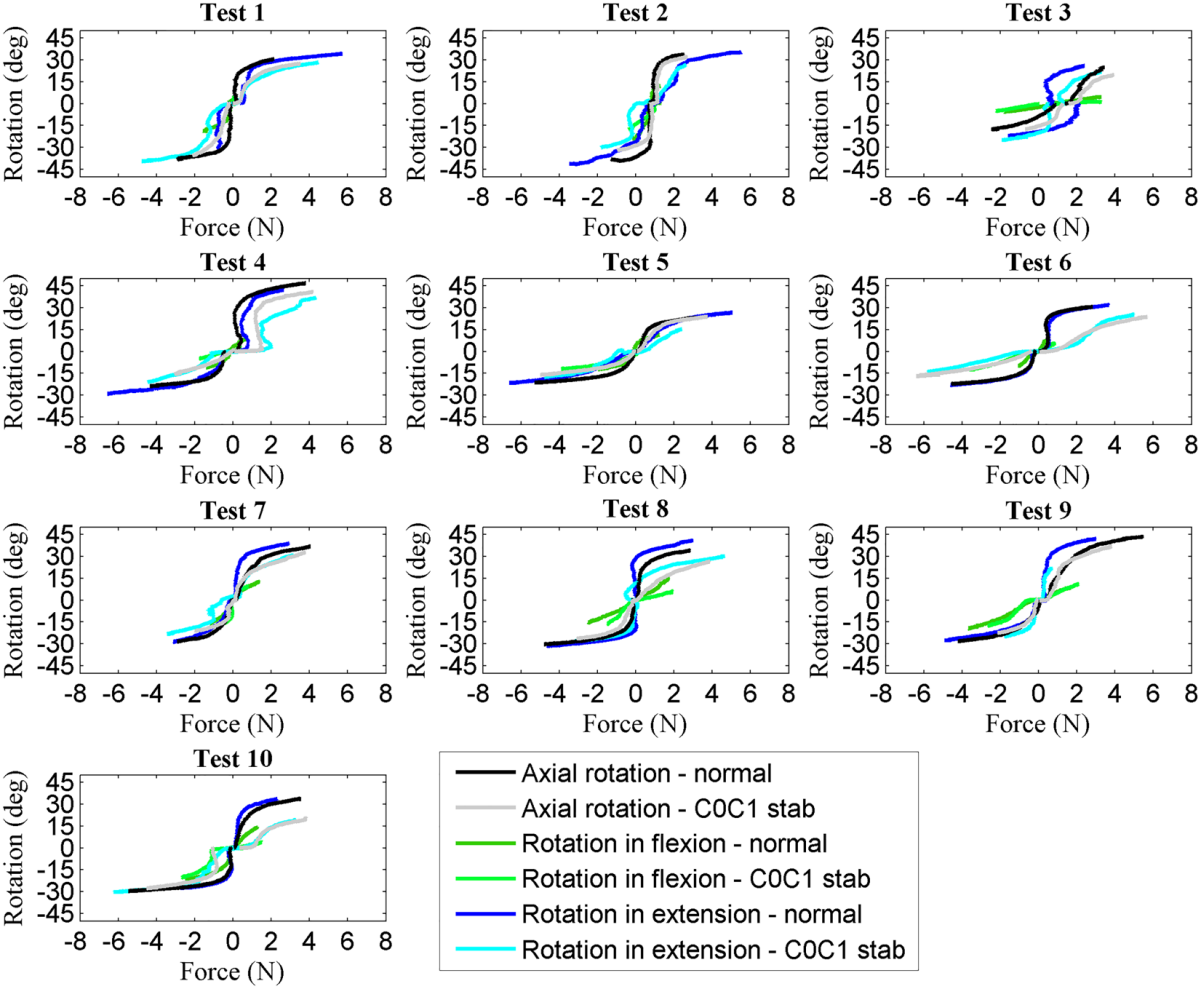
Amount of force and resultant range of movement for all ten specimens.

Figure 3 illustrates the amount of force applied and the resultant rotation + extension + contralateral lateral bending movement for all ten specimens without and with C0–C1 stabilization. Positive values indicate right rotation + extension + contralateral lateral bending and negative values indicate left rotation + extension + contralateral lateral bending.

During upper cervical right and left rotation + extension + contralateral lateral bending, the end ROM without C0–C1 stabilization was 35.1° ± 5.9° (with an average maximum force of 3.6N ± 1.3N) and 29.0° ± 6.5° (with an average maximum force of 4.1N ± 1.6N) respectively^13^. Following C0–C1 stabilization, there was a statistically significant reduction of right rotation in extension end ROM of 9.5° ± 4.9° (*p* = 0.007) present in all the specimens. After C0–C1 stabilization, there was a non-statistically significant reduction of 3.7° ± 4.9° (*p* = 0.32) (Table 2) in left rotation + extension + contralateral lateral bending. This ROM reduction in left rotation + extension + contralateral lateral bending was present in 7 out of the 10 specimens.

**Table 2:** Comparison between the ROM at different standardized forces and at end-range with the maximal force, and between the maximal force for non-stabilized and C0–C1 stabilization (C0C1 stab) configurations in rotation + flexion + ipsilateral lateral bending, rotation and rotation + extension + contralateral lateral bending.

	Right Rotation + flexion + ipsilateral lateral bending		Left rotation + flexion + ipsilateral lateral bending		Right Rotation + extension + contralateral lateral bending		Left Rotation + extension + contralateral lateral bending		Right Rotation		Left Rotation	
	Mean ± SD	*p* value	Mean ± SD	*p* value	Mean ± SD	*p* value	Mean ± SD	*p* value	Mean ± SD	*p* value	Mean ± SD	*p* value
1N_Normal	7.2° ± 4.1°	0.05 (n = 4)	10.4° ± 4.4°	**0.017** (n = 7)	24.5° ± 11.3°	**0.01** (n = 8)	20.5° ± 8.6°	0.11 (n = 10)	21.1° ± 11.3°	**0.011** (n = 9)	21.6° ± 8.6°	**0.012** (n = 8)
1N_ C0C1 stab	2.5° ± 2.1°		4.4° ± 3.7°		8.3° ± 8.5°		12.5° ± 10.8°		9.3° ± 8.5°		14.7° ± 7.7°	
2N_Normal	–	**-** (n = 1)	15.3° ± 4.4°	0.297 (n = 2)	30.1° ± 8.9°	**0.006** (n = 9)	24.7° ± 8°	0.15 (n = 7)	29.1° ± 9.1°	**0.005** (n = 10)	23.2° ± 6.5°	**0.018** (n = 7)
2N_ C0C1 stab	5.8° ± 7°		10.7° ± 5.3°		15.9° ± 7.8°		17.7° ± 10°		20.6° ± 10.3°		19.1° ± 9.6°	
F Max_Normal	1.3° ± 0.8°	0.76 (n = 10)	1.8° ± 0.9°	0.76 (n = 10)	3.6° ± 1.3°	0.94 (n = 10)	4.1° ± 1.6°	0.79 (n = 10)	3.4° ± 0.9°	0.093 (n = 10)	3.8° ± 1.4°	0.059 (n = 10)
F Max_ C0C1 stab	1.2° ± 1°		1.9° ± 1.3°		3.4° ± 1.3°		3.8° ± 1.6°		3.9° ± 0.7°		3.0° ± 1.8°	
ROM Max_Normal	9.8° ± 3.9°	0.09 (n = 10)	15.5° ± 5.9°	0.54 (n = 10)	35.1° ± 6°	**0.007** (n = 10)	29° ± 6.5°	0.32 (n = 10)	33.9° ± 6.6°	**0.005** (n = 10)	28° ± 6.9°	**0.013** (n = 10)
ROM Max_ C0C1 stab	6.7° ± 4.3°		13.6° ± 5.3°		25.7° ± 6.4°		25.3° ± 7.1°		28.5° ± 7°		23.6° ± 8.5°	

N: Newtons; F Max: Applied Force at End Range of Motion; ROM Max: End Range of Motion; SD: Standard Deviation; n: number of specimens for the comparison.

Significant values are in bold.

### Rotation + flexion + ipsilateral lateral bending mobility

Figure 3 illustrates the amount of force applied and the resultant rotation + flexion + ipsilateral lateral bending movement for all ten specimens without and with C0–C1 stabilization. Positive values indicate right rotation and negative values indicate left rotation.

During upper cervical right and left rotation + flexion + ipsilateral lateral bending, the end ROM without C0–C1 stabilization was 9.8° ± 3.9° (with an average maximum force of 1.33N ± 0.85N) and 15.5° ± 5.9° (with an average maximum force of 1.8N ± 0.9N) respectively. Following C0–C1 stabilization, there was a non-statistically significant reduction of right and left rotation + flexion + ipsilateral lateral bending end ROM of 3.1° ± 4.7° (*p* = 0.09) and 1.9° ± 3.5° (*p* = 0.54) respectively (Table 2). This ROM reduction was present in 7 out of the 10 specimens.

Table 2 shows the comparison between the ROM at different standardized forces and at end-range with the maximal force, and between the maximal force for non-stabilized and C0–C1 stabilization configurations in rotation + flexion + ipsilateral lateral bending, rotation, and rotation + extension + contralateral lateral bending. At the end ROM, right rotation + extension + contralateral lateral bending and both right and left rotations showed a statistically significant reduction of movement with C0–C1 stabilization. There were no statistical differences in the maximal forces applied without and with stabilization of C0–C1 in all directions.

## Discussion

To our knowledge, this is the first biomechanical study that analyzes the potential role of C0–C1 restriction of movement on the ROM of the different combined movements (rotation + flexion + ipsilateral lateral bending and rotation + extension + contralateral lateral bending).

The hypotheses of our study were only partly confirmed. As it was predicted, C0–C1 stabilization reduced UCS axial rotation in both directions and in right rotation + extension + contralateral lateral bending combination. However, there was not a statistically significant reduction in left rotation + extension + contralateral lateral bending and in both left and right rotation + flexion + ipsilateral lateral bending.

Apart from the specialized arthrokinematics of C1–C2, coupled movements associated to axial rotation have been proposed as an explanation for the large amount of rotation ROM in the UCS with intact alar ligaments^24^. UCS rotation associated with ipsilateral lateral bending and ventral flexion should tighten the contralateral alar ligament and limit UCS rotation prematurely^13^. However, rotation associated with contralateral lateral bending and extension should allow more UCS rotation ROM by delaying in the tightening of the alar ligaments^24^. Lorente et al. (2022)^13^ showed that the rotation + extension + contralateral lateral bending ROM was larger than the rotation + flexion + ipsilateral lateral bending combination.

Several studies have not frequently considered the C0–C1 segment when evaluating UCS rotation^2,8,17^. However, in vivo C0–C1 axial rotation to one side has been measured with values of 1.7° ± 1.5°^5^, and 2.5° ± 1.0°^6^ in samples with an average of 24.3 and 23.6 years respectively. Furthermore, up to 4.2° ± 1.8° has been reported in vivo by Dvorak et al.^25^ in young adults of 30 years. Values within these measurements have been quantified in other in vitro studies^10,26–28^. Previous in vitro studies applied up to 1.5 Nm in C0–C3 specimens^27^, but our goal was to replicate the load applied to patients, even though higher loads would have been possible without causing damage. The loads applied in our in vitro study might be even higher than the real loads on patients, as a previous study showed that therapy techniques tend to be more aggressive when applied in vitro^29^. Axial rotation of − 2°^30^ or even − 4°^31^ to the opposite direction of the C1–C2 rotation have been also reported at the atlanto-occipital joint in young adults.

Even with this minimal or paradoxical mobility in axial rotation, the arthrokinematics of C0–C1 seems to influence the degree of tightening of the alar ligaments and consequently to reduce UCS rotation. Thus, in our sample with an average of 74 years, the C0–C1 stabilization reduced the UCS ROM by 15.6% in axial rotation and 26.9% in right rotation + extension + contralateral lateral bending at end range of movement and also with 1N and 2N standardized forces. However, the reduction in left rotation + extension + contralateral lateral bending and both left and right rotation + flexion + ipsilateral lateral bending was not statistically significant. More studies are needed to validate if the UCS rotation + extension + contralateral lateral bending combination and the corresponding gliding of the C0 condyles would reduce the tightening of the alar ligament and allow for more UCS rotation^24^.

In our study, the average reduction of UCS in both directions were within the published values for C0–C1 rotation. However, in the rotation + extension + contralateral lateral bending combination, the average reduction of UCS rotation after C0–C1 stabilization was far beyond the normal values for C0–C1. In fact, C0–C1 stabilization resulted in a statistically significant reduction of right rotation + extension + contralateral lateral bending but not in left rotation + extension + contralateral lateral bending or both rotations in flexion.

This study presents the following limitations. The authors acknowledge that a larger sample may be necessary to demonstrate significant reduction in motion in all directions following C0–C1 stabilization as hypothesized. It is interesting to note that not all specimens showed the same mobility behavior. Three of the specimens in left rotation + extension + contralateral lateral bending, 1 specimen in left axial rotation and three specimens in both rotations in flexion did not reduce the ROM while some specimens demonstrated a 50% reduction of UCS rotation after C0–C1 stabilization. Inter-individual variability such as age-related degenerative changes, upper cervical anatomy variations such as variations in alar ligament orientation from dens to the occiput^32^, variability in the origin of the ligaments on the odontoid process, and an inconsistent atlantal portion of the alar ligament^4^ have been described in the literature and may have also impacted the results of this study and the left–right differences in the range of motion. Additional limitations of the present study relate to the mobilization procedure. The methodology used was original to this study making it challenging to compare the results with prior studies. The in vitro design used by this study allowed the stabilization of C2 to serve as a fixed point for movement reference. Also, the mobilization force was manually applied to simulate a clinical and physiological procedure in comparison to loading devices which may have been used in prior studies. Finally, the structures dissected before the applied movements^33^ and the presence of plates and sensors may alter the normal cervical spine conditions and also influence the results.

## Conclusion

The stabilization of C0–C1 reduced the upper cervical axial rotation in both right and left axial rotations and in right rotation + extension + contralateral lateral bending combination but not in left rotation + extension + contralateral lateral bending or both combinations of rotation + flexion + ipsilateral lateral bending.

## Data Availability

The datasets generated during and/or analyzed during the current study are available from the corresponding author on reasonable request.
